# Extracellular Vesicles as Biomarkers in Head and Neck Squamous Cell Carcinoma: From Diagnosis to Disease-Free Survival

**DOI:** 10.3390/cancers15061826

**Published:** 2023-03-17

**Authors:** Bojie Chen, Leanne Lee Leung, Xinyu Qu, Jason Ying-Kuen Chan

**Affiliations:** Department of Otorhinolaryngology, Head and Neck Surgery, Faculty of Medicine, The Chinese University of Hong Kong, Hong Kong, China

**Keywords:** extracellular vesicles, head and neck squamous cell carcinoma, exosome, miRNA

## Abstract

**Simple Summary:**

Extracellular vesicles are considered intracellular messengers that exchange proteins, lipids, various types of DNA and RNA, etc. They can be properly collected from many kinds of biofluids, such as blood and saliva. Compared to other substances obtained from liquid biopsy, such as cell-free DNA and circulating tumor cells, extracellular vesicles are a better choice to evaluate the disease since the genetic information is protected by their membranous structure, and thus they are more stable for detection. Extracellular vesicles contain less invasive property compared with other substances, and they are easier to collect from extraction with higher efficiency. Moreover, the roles of extracellular vesicles in multiple physiological and pathological processes are more well-known, entitling them to serve as biomarkers for certain cancers, including head and neck squamous cell carcinoma. This review aims to comprehensively present the current clinical trials at several key stages of tumor diagnosis and treatment, as well as their potential to dynamically monitor head and neck cancers during the whole clinical care process.

**Abstract:**

Head and neck squamous cell carcinomas (HNSCCs) arising from different anatomical sites present with different incidences and characteristics, which requires a personalized treatment strategy. Despite the extensive research that has conducted on this malignancy, HNSCC still has a poor overall survival rate. Many attempts have been made to improve the outcomes, but one of the bottlenecks is thought to be the lack of an effective biomarker with high sensitivity and specificity. Extracellular vesicles (EVs) are secreted by various cells and participate in a great number of intercellular communications. Based on liquid biopsy, EV detection in several biofluids, such as blood, saliva, and urine, has been applied to identify the existence and progression of a variety of cancers. In HNSCC, tumor-derived EVs exhibit many functionalities by transporting diverse cargoes, which highlights their importance in tumor screening, the determination of multidisciplinary therapy, prediction of prognosis, and evaluation of therapeutic effects. This review illustrates the classification and formation of EV subtypes, the cargoes conveyed by these vesicles, and their respective functions in HNSCC cancer biology, and discloses their potential as biomarkers during the whole process of tumor diagnosis, treatment, and follow-up.

## 1. Introduction to Extracellular Vesicles and Head and Neck Squamous Cell Carcinoma

### 1.1. Head and Neck Squamous Cell Carcinoma

For the disease condition, more than 90% of head and neck cancers are histologically squamous cell carcinomas (HNSCCs) [1]. HNSCC commonly encompasses the cancers in the mucosal epithelium of the nasal cavity, paranasal sinuses, oral cavity, pharynx, larynx, and salivary glands [2,3]. Cancers arising from different anatomical sites exhibit various incidences: the estimated age-standardized incidence for HNSCC was 4.84 for the lip and oral cavity, 2.59 for laryngeal cancers, and 1.35 for nasopharyngeal cancers [4]. The overall incidence is still growing and is anticipated to rise by 30% by 2030 [1]. HNSCC is also more prevalent in males in certain global regions, such as Hong Kong, India, Central and Eastern Europe, and America [1]. Its prevalence varies across countries and regions with different oncogenic factors: in Southeast Asia and Australia, the consumption of certain carcinogen-containing substances, such as tobacco and alcohol, are the leading risk factors of HNSCC [5]; in the USA and Europe, it is largely attributed to human papillomavirus (HPV) infection [6,7]. HPV-positive HNSCC is associated with carcinomas of the oropharynx. As for the HPV-negative group, they are usually located in the oral cavity and larynx, closely associated with a history of smoking [2]. The reason that the early detection of HNSCC is hard to achieve is partly due to the lack of effective screening strategies, except for opportunistic, careful physical examinations [2].

In some cases of HNSCC, a timely intervention helps to prevent premalignant lesions from developing into HNSCC. Oral premalignant lesions present as leukoplakia (white patches) or erythroplakia (red patches), which are relatively easier to be observed than pathological changes at other sites [2]. However, for most cases, the patients present at a relatively advanced stage, and up to 10% of them undergo metastasis without any premalignant history or lesions [8,9].

The five-year overall survival rate of HNSCC remains dismal: increasing from just 55% during 1992−1996 to 66% in 2002−2006 and staying stagnant since then, especially in older patients [1]. The primary reasons are considered to be the late diagnosis, the high tendency of recurrence or metastasis, and resistance to therapies [10]. Current multidisciplinary treatment for HNSCC is composed of three modalities: surgery, radiotherapy, and anti-cancer drug therapy [10]. The treatment strategies are decided by many factors. For HNSCC in the oral cavity, the first approach is usually surgical resection, potentially followed by adjuvant chemoradiotherapy (CRT); for HNSCC arising from the pharynx or larynx, the primary choice will shift to CRT in lower-volume diseases [9]. Except for early stage HNSCC, the majority of therapeutic strategies involve multiple modalities [9]. Since the advent of cisplatin and the development of cetuximab for the treatment of HNSCC, the diversifying repertoire of anti-cancer drugs has become significantly important in alleviating locally advanced/resistant/metastatic HNSCC (LA- or R/M HNSCC) [11]. For those unresectable, cisplatin-refractory recurrent or metastatic HNSCCs, the immune checkpoint inhibitor pembrolizumab has been approved as a first-line therapy [12,13]. The exploration of the underlying mechanism of the tumor characteristics of HNSCC can shed light on the identification of certain molecular biomarkers, which could aid in the discovery of occult lesions, monitoring of tumor growth, prediction of metastasis, and evaluation of prognosis.

### 1.2. Extracellular Vesicles

All types of cells possess membrane vesicles, known as extracellular vesicles (EVs), not only in normal homeostatic status but also in pathological conditions as well. EVs used to be considered cellular wastes, but currently they are proving to be a pivotal approach to understanding intercellular communications [14]. Tumor cells, immune cells, epithelial cells, and other complex components of the tumor microenvironment (TME) could be the source of EVs [15].

**Figure 1:**
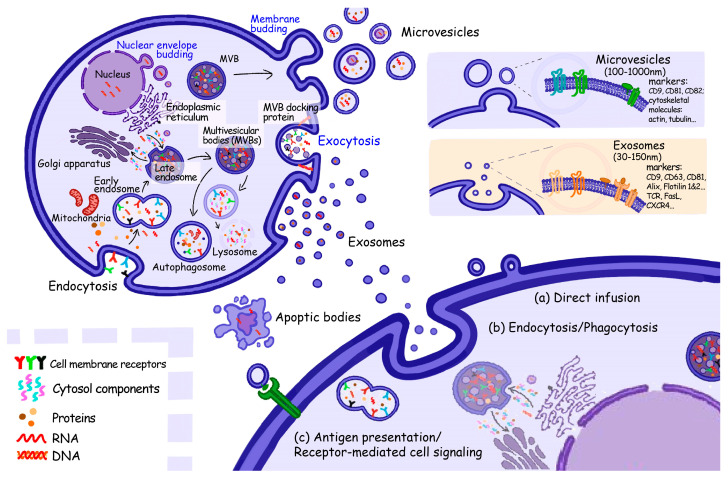
Biogenesis of EV subtypes and their transporting mode. Early endosomes sprout inwards from the cell membrane to form intraluminal vesicles, engulf intracellular components (such as RNAs, proteins, and other metabolites), transform into multivesicular bodies and then release exosomes into extracellular space. As for microvesicles, they bud from plasma membrane, and accepted by recipient cells via (**a**) direct fusion, (**b**) phagocytosis, (**c**) receptor-mediated or not endocytosis. EVs could be identified and extracted by liquid biopsy as cell-free DNA and circulating tumor cells.

EVs are divided into several subgroups by their origins, diameter, and surface protein markers, which mainly include exosomes (originating from the endosome, 30−150 nm) [16], microvesicles (MVs) (from the plasma membrane, 100−1000 nm) [17] and apoptotic bodies (from the plasma membrane, 50–5000 nm) (Figure 1) [18]. The classic dichotomy of EVs used to be size-dependent, but a recent expert consensus has placed increasing emphasis on the biogenesis and different molecular contents of EVs, which probably determine their sizes [19]. Exosomes possess relatively small volumes and are thought to participate in endosome biogenesis [20]. Early endosomal membranes sprout inwards to form intraluminal vesicles (ILVs), and ILVs are conveyed by multivesicular bodies (MVBs) during the endosomal maturation. After MVBs move towards the plasma membrane and then fuse with it, MVBs will consequently release exosomes into the extracellular space. The remaining MVBs will be lysed and degraded by lysosomes and hydrolytic enzymes [21,22]. In the late 1980s, exosomes were originally thought to be cell waste, but increasing research has documented the implications of exosomes in intercellular communication and immune regulations, and it has gradually been accepted that exosomes serve in numerous physiological and pathological processes rather than just in certain cell types [23]. MVs are generally larger than exosomes. The formation of MVs is greatly influenced by plasma membrane bending and budding during endocytosis, with its underlying mechanism inconclusive [24]. It used to be called “platelet dust”, since it was found to originate from the serum and participate in blood coagulation [25]. As more was learned about the features, certain MVs from cancer cells, called oncosomes, were proven to trigger or activate related pathways, which likely influence TME and metabolic phenotypes [26]. Besides the cargoes that are conveyed by exosomes and MVs, including DNA, RNA, proteins, and lipids, organelles are specific cargoes only delivered by apoptotic bodies [26].

**Figure 2:**
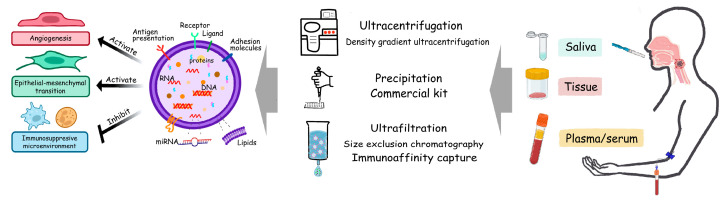
EV cargoes and the clinical application of EV-miRNA during HNSCC diagnosis and treatment processes. The exact influences caused by EVs depend on their cargoes. Oncogenic EVs activate the vasculature, promote epithelial–mesenchymal transition, facilitate formation of pre-metastatic niche, protect tumor cells from immune supervision and realize many other functions. Based on current technologies, EVs could be detected and extracted from saliva, blood, urine and tissue samples. Much evidence supports the great significance of EV-miRNAs in the diagnosis, progression, prognosis prediction and treatment evaluation of HNSCC. The miRNAs shown in bold (e.g., BART7-3p) are EBV-derived.

EVs from different origins consist of various components and present with cell-type-specific proteins. Even from the same cell of origin, the contents of EVs vary with different stimuli and microenvironments, and consequently, they have corresponding fates and functions. Since EVs were discovered, there has been the notion that EVs could potentially contribute to oncogenesis, metastasis, resistance to chemotherapy, and therefore, consequently serve as tumor biomarkers. Rapid detection has become a hot spot in cancer research (Figure 2). However, there are still many barriers to realizing clinical transformation to the detection of biomarkers of EVs, especially in the common widely used isolation methods. For example, with ultracentrifugation, the representative traditional EV isolation method, the main problems are membrane damage and soluble protein contamination with the EV particles [27]. In density gradient ultracentrifugation, a recent common EV purification method, the isolated EV pellets yield a better quality product, but it is time consuming and has low efficiency [28,29]. The precipitation or commercial kit is relatively efficient at isolating EVs, but reports have mentioned that there are miRNA contaminations and a decreased EV subpopulation enrichment [28,29]. For the other EV isolation methods, due to the small sample size for clinical applicability or validation, the detection methods of ultrafiltration, size exclusion chromatography, and immunoaffinity capture have not been put into wide clinical use [28,30]. The leading method to detect EVs or related biomarkers in this field, such as DNA, RNA, and proteins conveyed by EVs, involves size-based and immunoaffinity-based detection. The principle is the combination method that accomplishes the attachment of the “probe”, such as the aptamers of specific EV membrane proteins or EV marker-bound magnetic beads, for detecting EVs or their intravesicular cargoes, to condense them together by the physical or chemical filters [30,31,32,33].

Extracellular vesicles are also called intercellular messengers since they are found to participate in the tumor microenvironment [34]. With EVs, the mutual communication between parent tumor cells and other types of cells is established and then amplified, for instance, paracrine interactions between tumor cells, immune cells (e.g., B-lymphocytes, natural killer cells [35]), epithelial cells, and stromal cells; juxtacrine signaling between tumor cells and tumor-infiltrating T-cells (TILs); and auto-communications between tumor cells and the exosomes released by themselves [36,37]. In the TME, exosomes released by tumor cells re-program some effector cells, such as cancer-associated fibroblasts (CAFs) and mesenchymal stem cells (MSCs), and consequently, they are pro-tumorigenic by promoting tumor growth and metastasis [22,38,39]. As major components of the TME, CAFs and exosomes promote the switch of the metabolic phenotype of cancer cells from mitochondrial oxidative phosphorylation to glycolysis and glutamine-dependent reduction carboxylation [38]. At the level of microRNAs (miRs), the expressions of miR-21, miR-378e, and miR-143 in exosomes derived from CAFs in breast cancer are higher than exosomes from normal fibroblasts, which increases the stemness, epithelial–mesenchymal transition (EMT), invasion and migration of these tumor cells [39]. Additionally, other connective cells, such as MSCs, also play a vital role in the TME. The promotion of tumor growth by MSC-derived EVs, similar to MSCs, was supposed by Zhu et al. [40]. Years later, current studies have been trying to make full use of their paracrine signaling pattern in order to use them as vesicles to deliver therapeutic RNA fragments [41].

Tumor-secreted EVs have their own specific integrin profiles that enable them to move to target organs, and they are vital participants in tumor metastasis [42]. They upregulate pro-inflammatory S100 molecules in the target organs, and their close interactions with metastatic tumor cells promote pre-metastatic niche development. For instance, melanoma-secreted exosomes promote vascular leakiness and inflammation, a hallmark of pre-metastatic niche formation, by upregulating factors such as S100a8, S100a9, and TNF-α [43]. Additionally, EV-RNAs from normal cells can also make the behaviors of malignancies more aggressive. In HCC cells, adipocytes release exosomal circRNA to reduce DNA damage of tumor cells by targeting miR-34a, resulting in metastasis in vivo [44]. Overall, EVs deliver cargoes to stimulate the vasculature and fuse with the parenchymal cells where pre-metastatic niches are colonized to facilitate tumor metastasis.

The regulation of EVs in the immune system, reducing T-cell activity, promotes tumor immune escape via programmed cell death protein-1 (PD-1) and its ligands via a programmed-death-ligand-1 (PD-L1)-related pathway. In oral squamous cell carcinoma (OSCC) patients, mitochondrial Lon, a multiple-function protein that is in charge of protein quality control, metabolism, mitophagy, and stress response, stimulates the cGAS-STING-TBKI pathway and induces interferon (IFN), consequently upregulating PD-L1 [45]. Additionally, EVs secreted by Lon can transport oxidized mitochondrial DNA and PD-L1, which inhibits T-cell activation in the TME and contributes to tumor immune escape [45]. In a similar way, exosomes found in lung cancer containing PD-L1 suppress IFN-γ secretion by Jurkat T-cells and impair immune functions by reducing cytokine production and inducing apoptosis in CD8+ T-cells [46].

The resistance of chemoradiotherapy in certain malignant tumors is enhanced by EVs through multiple mechanisms. In gastric cancers, exosomal miR-155-5p has been proven to induce chemo-resistant phenotype transformations by inhibiting GATA-binding protein 3 (GATA3) and tumor protein p53-inducible nuclear protein 1 (TP53INP1) [47]. As for ovarian cancer, exo-miR-21 mediates the interactions between stromal tissue and tumor cells and potentiates resistance to chemotherapy [48]. Radiotherapy (RT) is the primary treatment for nasopharyngeal carcinoma (NPC) and radio-resistance prevails as a dominant problem in clinical practice. EVs derived from Epstein–Barr-virus (EBV)-encoded latent-membrane-protein-1 (LMP1)-positive NPC cells significantly transmitted LMP1, which could activate the p38 MAPK signaling pathway to induce recipient tumor radio-resistance [49].

With the extensive study of EVs, their potential to work as biomarkers or drug carriers has been gradually explored. There are several merits to EVs as the carriers of therapeutic cargo, since they are naturally compatible with the body, relatively stable in circulation, ideally permeable through various membranes and barriers, and tissue- or cell-type-specific [50]. The targeting ligands are engineered to fuse with EV transmembrane proteins, which make up plasmid fusion constructs and fulfill the specificity of EVs. Therapeutic contents in EVs can be loaded in two modes: endogenous or exogenous. Endogenous loading is based on the overexpression of certain cargo in targeting source cells, and EVs are collected with the drug, whereas exogenous loading collects drug-free EVs [50]. Increasing numbers of trials have been implemented and verified the feasibility of EVs’ therapeutic applications [51,52,53,54]. Since the presence, quantity, and molecular content of EVs are believed to reflect the oncogenesis, progression, and malignant behaviors of tumors, many studies have verified their predictive values in diagnostic or prognostic strategy by in vivo and in vitro cancer biology experiments in different tumors, including cancers of the breast [55], lung [56], gastrointestinal tract [57,58], hepatobiliary system [59,60], prostate [61], head and neck [62,63,64]. The type of sample is determined by the lesion site, which leads to higher cost-efficiency, less invasion, and easier real-time measurement; for OSCC, plasma and saliva are the primary choices [62].

The endeavor to consider EVs as biomarkers in the clinical practice of HNSCC is still in its infancy, but recently, several researchers have supported that EVs with various molecular components have roles in tumorigenesis, progression, metastasis, recurrence, and the alteration of the TME [62,63,64].

## 2. Overview of Biomarkers in HNSCC—cfDNA, Circulating Tumor Cells, EVs

A biomarker can reflect specific biological features and molecular activities of certain diseases, such as in malignancies. Similar to α-fetoprotein in hepatic cell carcinoma [65], prostate-specific antigen in prostate cancer [66], or human chorionic gonadotropin-β in gestational trophoblastic tumors [67], squamous cell carcinoma antigen (SCC-Ag) is a potential biomarker for HNSCC, whereas an elevated level of SCC-Ag also suggests other diseases besides HNSCC, such as skin inflammation, autoimmune diseases and malignancies [68,69,70]. Given the lack of a sensitive and specific biomarker for HNSCC, researchers have continued exploring more potential biomarkers for HNSCC for early diagnosis, to predict therapeutic responses, to facilitate disease surveillance and to ultimately improve patients’ survival.

The biomarkers can be extracted from several kinds of patient samples, including tissue, plasma, saliva, or other biofluids. Tissue samples, which have the highest diagnostic value, are obtained by aspiration biopsy or other invasive procedures, which probably imposes greater mental stress on patients. Therefore, liquid biopsy and appropriate biomarkers are a better choice in light of the potential high efficiency and low invasiveness [71]. Additionally, their emphasis on the dynamic evolution of diseases partly alleviates the spatial and temporal heterogeneity in tumor tissue samples, which provides a general guide for treatment decision making and benefits patient monitoring and follow-ups [71]. The common liquid biopsy analytes include cell-free DNA (cfDNA), circulating tumor cells (CTCs), circulating RNAs, EVs, other proteins, and metabolites.

### 2.1. Cell-Free DNA

Cell-free DNA (cfDNA) is an extracellular fragment of DNA present in the body fluid, which was first discovered in the circulatory system by Mandel and Metais in 1948 [72]. Subsequently, circulating tumoral DNA (ctDNA), with obviously specific double-strand instability, was found in cfDNA in cancer patients’ DNA by Stroun et al. [73]. Besides tumor-originated, cfDNA, cell-free fetal DNA and cell-free circulating mitochondrial DNA, which have their own characteristics, have also been found. There are many ways for DNA fragments to be released from the inside of cells and finally into circulation or other biofluids, including through necrosis, apoptosis, active DNA release, neutrophil extracellular traps, EV transportation, erythroblast enucleation, and exogenous sources [74]. The first report that demonstrated the predictive values of cfDNA on tumor prognosis was published in the 1970s [75]. It showed that a higher cfDNA level indicates tumor metastasis, and it decreases after the radiation therapy. However, persistent or unchanged cfDNA levels reflect an ineffective treatment and serve as a sign of poor prognosis [75].

During the last few decades, liquid biopsy technology has been developed and its clinical workflow improved. cfDNA can be used for tumor progression, with plasma samples preferred over serum since there is too much non-tumor-derived cfDNA generated during the blood clotting by leukocyte lysis during serum sample preparation [71].

Recently, an increasing number of clinical studies have compared liquid-biopsy-based cfDNA detection and tumor-tissue-based aspiration, showing the distinctive advantages of liquid biopsies over tumor-tissue-based aspiration: less invasiveness, less heterogeneity, great reliability, and more rapid turnaround time [76,77,78,79]. Therefore, cfDNA screening is considered in the surveillance of tumor progression, tracing cancer metastasis, and guiding the selection of treatment strategies.

The clinical samples collected for cfDNA detection in HNSCC patients are mainly blood samples, including plasma and serum, but there are also several trials using saliva, which is more easily acquirable but requires more advanced detection methods [79,80,81]. Comparing HNSCC patients and healthy controls, the overall copy number of cfDNA in HNSCC patient samples is obviously higher than in healthy controls, which emphasizes the screening function and diagnostic value of cfDNA [82,83]. Moreover, a higher total copy number of cfDNA or a greater level of TP53 expression also indicate more aggressive tumor behaviors, such as LNM and advanced stage, in HNSCC patients [82,83]. Intriguingly, in HPV-related HNSCC cases, especially in oropharyngeal squamous cell carcinoma (OPSCC), the quantification of cf-HPV-DNA displays high specificity, positive and negative likelihood ratios, and a diagnostic odds ratio when distinguishing HPV-related OPSCC from normal controls and predicting the post-surgery recurrence [84].

However, so far there are certain limitations to reaching a conclusion about how the detection of cfDNA serves as an efficient tumor-screening tool. Most of the current studies are from North America and Europe, which may have biases by not including other regions, particularly in relation to HPV-negative OPSCC. Another problem is the lack of unified detection method for cfDNA, which is a common obstacle to utilizing a novel technique in clinical practices.

### 2.2. Circulating Tumor Cells, CTCs

CTCs are tumor cells shedding from the primary cancer site into blood circulation or the lymphatic system, which might extravasate and develop into metastatic sites [85]. Since the concept of CTCs was first proposed, many studies have shown that they are precursors of metastasis in certain cancer types [86,87,88,89]. Therefore, they are also considered to be an ideal tool to monitor tumor progression and therapeutic effects, which are of value in prognosis prediction and offer a choice of treatment strategy to avoid inappropriate surgical intervention or adjuvant drugs.

CTCs can be classified into several subtypes according to multiple factors, including epithelial markers, cell morphology, and apoptotic status. Epithelial-originated CTCs are distinguishable from other phenotypes due to the expression of EpCAM and cytokeratin [90,91,92]. Since mesenchymal CTCs lack cytokeratins and CD45, they are less likely to be isolated from the blood, which also makes them more resistant and more prone to metastasis [93,94]. Apoptotic CTCs result from the process of programmed cell death, the level of which provides a reference for evaluating treatment effects [95,96].

CTCs are few in quantity, up to 10 CTC per milliliter of whole blood compared to a few million white blood cells and a billion red blood cells [97]. Their half-life is less than 3 h, which can also be influenced by external conditions [97]. To achieve a high sensitivity, specificity, and reproducibility, CTC-detecting technologies keep improving, which are broadly classified into biological methods, physical methods, and hybrid methods [98,99,100]. As for the biological mechanism, the isolation of CTCs is based on the antigen–antibody combination in which the common markers include EpCAM as a cancer stem cell marker, and HER2 and PSA as the targets for breast and prostate cancer, respectively [90,91]. The CellSearch system, an immunomagnetic assay with magnetic nanoparticle-based separating technology, is the cleared U.S. Food and Drug Administration (FDA) biological methodology for CTC enumeration [101]. Physical methods generally capture CTCs by various size filtrations, and as the representative, the Parsortix^®^ PC1 system was cleared by the FDA in May 2022 [102]. However, these technologies have their own drawbacks that may have some biases, for instance, the biological extraction of CTCs requires a sufficient expression of the known and selected marker on the cell surface, and physical filtration would probably overlook the other blood components whose diameters and properties are close to those of CTCs [92,100].

Tinhefer et al. and Garrel et al. successfully found the presence of CTCs in HNSCC, which was related to shorter disease-free survival (DFS) [103,104]. In addition, the number of CTCs extracted from peripheral blood could also serve as a predictor of tumor prognosis and a dynamic tracking tool to assess tumor tolerance to personalized therapy [89,105]. CTCs have been explored in patients with relapsing non-operable or metastatic HNSCC [103]. One study reported that the analysis of a patient’s peripheral blood samples collected on day 0 (D0), D7, and D21 after the intervention of chemotherapy and cetuximab dynamically reflected the tumor response to the therapy by CTC kinetics within the first week of treatment, and increasing or stable counts of CTCs suggested poor therapeutic effects and earlier tumor progression [103].

Currently, there are still some limitations and drawbacks for CTCs when acting as biomarkers in HNSCC. HNSCC is an epithelial-originated cancer, but in reality, EpCAM is not always present in every CTC derived from HNSCC cases due to the transition between epithelial and mesenchymal characteristics, which results in the high specificity but low sensitivity of the current detection methods [90,91,100,106]. More innovations in CTC enumeration techniques are needed, such as a new Epithelial ImmunoSPOT (EPISPOT) assay by adding anti-CD45 antibodies to alleviate the influence of leukocytes and enrich the number of CTCs for detection [103].

### 2.3. Extracellular Vesicles, EVs

Compared to CTCs, the content of EVs in circulation is relatively abundant. Many physicochemical techniques have been developed to isolate and extract EVs from body fluids, including ultracentrifugation, size-based filtration, polymer precipitation, and microfluidics-based isolation [107,108]. Additionally, antigen–antibody-interaction-based immunoaffinity methods have also been adapted to isolate EVs, where magnetic microbeads coated with specific antibodies significantly bind to certain surface markers and extract greater than 10-times-higher quantities of EVs than ultracentrifugation [107,109].

Saliva is an ideal sample type for liquid biopsy due to its easy collection and non-invasiveness, with multiple studies making full use of the components in saliva to screen for malignancy, evaluate progression and prognosis. A study focusing on the values of HPV-16 DNA in distinguishing tumor patients from healthy controls showed a relatively low sensitivity (about 50%) of saliva-based detection and stated that the sensitivity of HPV-16 DNA prediction can be improved by the combination of saliva-based and plasma-based samples (reaching up to 69.5%) [110]. However, as for EVs in the saliva sample, since they were first discovered and successfully isolated, a growing number of studies have demonstrated the advantages of saliva samples as the medium for EV detection; a high number of EVs in saliva makes it simple to collect and present significant differences between patients and the normal population [111,112,113]; the samples are much easier to store and require fewer procedures to complete the identification and quantification of EVs due to fewer protein components compared to plasma [114].

Different subtypes of EVs have shown value in tumor diagnosis. Zlotogorski-Hurvitz et al. successfully established computational-aided models for the Fourier-transform infrared spectra of salivary exosomes from OSCC and indicated that it exhibited both high sensitivity and specificity for diagnosis [115]. Functional signatures of the protein conveyed by small salivary EVs were developed to predict OSCC with the assistance of the sequential window acquisition of all the theoretical mass spectra and a subsequent bioinformatics analysis [116]. The investigations into MVs in HNSCC have been few, and their origins remains controversial. Kim disclosed that the samples collected from clinical patients and cell lines of OSCC contained FasL, a member of the tumor necrosis factor family, which participates in tumor progression and metastasis [117]. FasL-positive MVs can theoretically enhance the immune suppression and apoptosis of activated immune cells, and MVs can maintain a relatively high level in the serum of OSCC patients, where they may act as an indicator of the tumor stage [117,118].

There is no widely recognized biomarker for HNSCC in clinical practice so far, which is probably restricted by the current detection techniques, insufficient data support, and regional disparity due to economic and medical strength, and EVs are expected to lead to a breakthrough in this field.

## 3. EV Cargoes and Their Instructive Effects in HNSCC

In tumor-derived EVs (TDEs), the cargoes conveyed by EVs could be DNA molecules, different kinds of RNAs, proteins, and even artificially synthesized pharmacological compounds, which have different functions in numerous vital pathways in HNSCC biology.

### 3.1. DNA

Double-stranded DNA (dsDNA) delivered by EVs in circulation possesses the same features and oncogenic gene mutations as the primary tumor DNA [119]. KRAS and TP53 expression have both been found in circulating EVs and pancreatic cancerous cells, which confirm their potential to be tumor biomarkers [119,120]. However, the genetic information about the whole genome sequence carried by EV-DNA seems to be limited when compared to formalin-fixed paraffin-embedded (FFPE) tumor samples, since the efficiency of DNA extraction is be significantly impacted by the various methods of EV isolation and DNA extraction [121]. Therefore, so far, it is not appropriate to select EV-associated DNA alone as the marker for tumor progression [121]. However, novel research in this field is focused on the detection of viral DNA. As a pivotal transmitter in intercellular communication, EVs play a significant role in tumor cells and virally infected cells in their microenvironment [122,123]. Viruses can also make use of EVs to escape from immune attack and disseminate infections, which has also been confirmed by several reports that EVs containing EBV-originated DNA and miRNA could be detected in the peripheral blood samples of NPC patients [122,123]. More than 90% of NPCs in endemic regions are associated with EBV, a γ-Herpes virus with long, linear, double-stranded DNA genomes [124]. EBV infection is commonly asymptomatic, but when activated, EBV may present as inflammation, such as mononucleosis [125,126], and induce the development of NPC and other malignancies, including Burkitt’s lymphoma and NK/T-cell lymphoma [127,128]. In EBV-infected NPC patients, the virus persists as a type II latency [129], and the expression of certain genes, including EBNA1, LMP1, LMP2A, EBER 1 & 2, and BARTS, can be detected [130]. The expression of LMP1, LMP2A, BARTS, and EBV DNA have been found in and delivered by EVs [49,130,131], which is a potential indicator of viral infection and tumor progression.

### 3.2. RNA

Ribonucleic acids (RNAs) are classified by many methodologies, such as by function, including messenger RNA (mRNA), transfer RNA (tRNA), and ribosomal RNA (rRNA) [132]; or by length, including small RNA (shorter than 200 nt; e.g., some rRNA, tRNA, microRNA (miRNA), and small interfering RNA (siRNA)) [133] and long RNAs (greater than 200 nt, e.g., long non-coding RNA (lncRNA) and mRNA) [134]. There are also some special RNAs with unique structures, such as double-stranded RNA and circular RNA (circRNA) [135].

Ma et al. investigated the mediation of cell-to-cell communication by exosomal shuttle RNA; they also verified the regulation of tumor growth by miRNAs (exo-miR-17/20) by interacting with target downstream proteins, such as IL-8, CXCL1, CK8 and α-ENO [136]. Despite the lack of a coding function, miRNAs can induce mRNA degradation and greatly influence protein expression at the post-transcription level [137]. It has been suggested that an active sorting mechanism of miRNAs into EVs exists that confers differing EV-miRNA regulations of tumor behaviors [137]. MiRNA can be extracted from many kinds of biofluids, such as saliva. Gallo et al. demonstrated that the majority of miRNA was enveloped by exosomes, and could be detected in a just small amount of saliva; additionally, it more stably exists in the saliva with the protection by a bi-layer membranous structure, which facilitates its development as a biomarker for HNSCC [138,139]. Distinguishable levels of oncogenic miRNA are anticipated to facilitate the early detection of HNSCC, which has been illustrated by numerous studies, as shown in Table 1. There were also some studies that analyzed particular miRNAs rich in HPV(+) and HPV(-) HNSCCs, respectively. Ludwig et al. reported that miR-205-5p was exclusively expressed in HPV(+) oropharyngeal squamous cell carcinoma (OPSCC) cell lines (UD-SCC-2, UM-SCC47, UPCI: SCC90)-derived exosomes, and miR-1972 was extraordinarily presented in HPV(−) cell lines (PCI-13 and PCI-30) [140]. When it comes to EBV, it was confirmed that some viral miRNAs were selectively packaged into virus-derived exosomes so that the viral infection could be promoted and transferred [141]. The diverse effects of miRNA in each stage of HNSCC diagnosis and treatment have been overviewed, and a literature review based on the biological source of the samples of the clinical patient cohort is shown in Table 1.

LncRNA is a class of RNA longer than 200 nucleotides but without protein-coding capacity [134]. EVs could envelop and transport lncRNA from tumor cells to target cells, consequently influencing the expression of miRNA and changing the tumor microenvironment (TME) [142]. One of the most representative lncRNAs was HOTTIP, and it caused various changes according to its origin: the M1 macrophage-derived exosomal lncRNA HOTTIP proved to strongly suppressed tumor progression via the upregulation of the TLR5/NF-κB pathway [143]; however, when it was secreted by cancer stem cells in OSCC, it then promoted M2 macrophage polarization, inhibited CD4+ T-cell proliferation and IFN-γ production, and facilitated tumor progression and immunosuppression via the LAMC2-mediated PI3K/AKT pathway [144].

CircRNA possesses a covalently bonded structure with a closed loop and without 5′ caps or 3′ poly(A) tails. Due to its relatively stable structure, wide distribution, and tissue specificity, it is superior when selected to act as molecular diagnostic marker and drug treatment target [145]. There are, however, few reports on circRNA detection in HNSCC screening. In OSCC-derived EVs, circRNA proved to be stably present in abundance [146], to be oncogenic through the upregulation of certain circRNAs in HNSCC, such as circCORO1C via the let-7c-5p/PBX3 axis [147], circRNA MTCL1 by inhibiting C1QBP ubiquitin degradation and beta-catenin activation [148], circPARD3 in autophagy through the PRKCI-Akt-mTOR pathway [149], and circPVT1 via the p53/YAP/TEAD transcription-competent complex [150].

**Table 1:** The performance of EV-miRNA in the diagnosis, progression, prognosis prediction and treatment evaluation of HNSCC.

First Author	Year	Cancer Type	RNA	Sample Type	Grouping	EVs Isolation Method	RNA Detection Method	Clinical Use	Ref.
Wang	2014	LSCC *	miR-21	Serum	LSCC (*n* = 52); benign laryngeal disease (*n* = 49)	Precipitation	qRT-PCR	Diagnosis, prognosis	[151]
Li	2016	OSCC *	miR-21	Serum	OSCC (*n* = 108); healthy control (*n* = 108)	ExoQuick kit (SBI)/precipitation	qRT-PCR	Progression (metastasis)	[152]
Ye	2016	NPC *	miR-24-3p	Plasma	NPC (*n* = 65); healthy control (*n* = 20)	Ultra-centrifugation (UC)	qRT-PCR array, qRT-PCR	Prognosis	[153]
Langevin	2017	HNSCC *	miR-486-5p, miR-486-3p, miR-10b-5p, miR-31	Saliva	HNSCC (*n* = 11); healthy control (*n* = 9)	UC	Sequencing, ddPCR	Diagnosis	[154]
Ramayanti	2019	NPC	BART7-3p, BART9-3p, BART13-3p	Serum	NPC (*n* = 39); healthy control (*n* = 33)	SEC Sepharose CL-2B column	LightCycler stem-loop RT-PCR	Diagnosis	[155]
Momen-Heravi	2018	OSCC	miR-21, miR-27, miR-412-3p, miR-512-3p	Saliva	OSCC (*n* = 24); healthy control (*n* = 10)	ExoQuick kit (SBI)/precipitation	qRT-PCR and TaqMan miRNA assay	Diagnosis	[156]
Bao	2018	NPC	miR-23a	Tissue, serum	NPC (*n* = 150)	UC	qRT-PCR	Progression (metastasis), prognosis	[157]
Gai	2018	OSCC	miR-512-3p, miR-412-3p, miR-302b-3p, miR-517b-3p, miR-21, HOTAIR	Saliva	OSCC (*n* = 21); healthy control (*n* = 11)	Precipitation	qRT-PCR array, qRT-PCR	Diagnosis	[112]
Lu	2018	NPC	miR-9	Plasma	NPC (*n* = 110); healthy control (*n* = 60)	UC	qRT-PCR	Diagnosis, prognosis	[158]
Xie	2019	OSCC	miR-101-3p	Tissue	OSCC (*n* = 36, tumor tissue vs. adjacent normal tissue)	UC	qRT-PCR	Progression (stage)	[159]
Qin	2019	HNSCC	miR-196a	Plasma	HNSCC (*n* = 74); healthy control (*n* = 30)	ExoQuick kit (SBI), precipitation	qRT-PCR	Diagnosis, prognosis, treatment response (cisplatin)	[160]
Sun	2019	OSCC	miR-382-5p	Cocultured tissue	47 OSCC patients (CAF-rich vs. CAF-poor)	Precipitation	qRT-PCR	Progression	[161]
Mayne	2020	OPSCC *	11-miR (ratio) panel	Serum	OPSCC (*n* = 40); GORD (*n* = 20); healthy control (*n* = 20)	Precipitation	Microarray	Diagnosis	[162]
Wang	2020	OSCC	miR-210-3p	Tissue	OSCC (*n* = 80); healthy control (*n* = 7)	UC	qRT-PCR	Progression (stage)	[163]
He	2020	OSCC	miR-24-3p	Saliva	HNSCC (*n* = 49); healthy control (*n* = 14)	ExoQuick kit (SBI), precipitation	Microarray, qRT-PCR	Diagnosis	[164]
Shuang	2022	LSCC	miR-27a	Plasma	LSCC (*n* = 62); healthy control uk	ExoQuick kit (SBI), precipitation	qRT-PCR array, qRT-PCR	Diagnosis, prognosis	[165]
Yin	2021	NPC	miR-433-3p	Tissue	LSCC (*n* = 13, tumor tissue vs. adjacent normal tissue)	UC	qRT-PCR	Progression (stage), prognosis	[166]
Tong	2020	HNSCC	miR-9	Tissue	HNSCC (*n* = 303)	UC	qRT-PCR	Prognosis, treatment response (RT *)	[167]
Jiang	2021	NPC	miR-134-5p, miR-205-5p, miR-409-3p	Plasma	NPC (*n* = 16); healthy control (*n* = 5)	Precipitation	Nanoparticle tracking analysis (NTA) and qRT-PCR	Diagnosis	[168]
Chen	2021	OSCC	miR-155, miR-21, miR-126	Serum	OSCC (*n* = 35); healthy control (*n* = 11)	Exosome Isolation Kit	Microarray, qRT-PCR	Progression (stage), prognosis	[169]
Liu	2021	NPC	miR-181a	Tissue	NPC (*n* = 134)	UC	qRT-PCR	Progression (metastasis, staging)	[170]
Bigagli	2021	OSCC	miR-210	Plasma	OSCC (*n* = 30); healthy control *(n* = 14)	Precipitation	qRT-PCR	Diagnosis, prognosis	[138]
Panvongsa	2021	HNSCC	miR-491-5p	Plasma	HNSCC (*n* = 73); healthy control (*n* = 20)	ExoQuick kit (SBI), precipitation	miRNA microarray analysis, qRT-PCR	Diagnosis, prognosis	[171]
Yu	2022	LSCC	miR-15b-5p	Cocultured tissue	LSCC (*n* = 16, tumor tissue vs. adjacent normal tissue)	ExoQuick kit (SBI), precipitation	qRT-PCR	Progression (LNM *)	[172]
Jiang	2022	NPC	miR-197.3p	Tissue	NPC (*n* = 40, with good vs. poor RT effects)	ExoQuick kit (SBI), precipitation	qRT-PCR	Treatment (RT *)	[173]
Lou	2022	OSCC	miR-626	Serum, tissue	OSCC (*n* = 65, tumor and serum; *n* = 10, tumor tissue vs. adjacent normal tissue)	UC	qRT-PCR	Prognosis	[174]

* LSCC, laryngeal squamous cell carcinoma; OSCC, oral squamous cell carcinoma; NPC, nasopharyngeal carcinoma; HNSCC, head and neck squamous cell carcinoma; OPSCC, oropharyngeal squamous cell carcinoma; RT, radiotherapy; LNM, lymph node metastasis.

### 3.3. Protein

There is a broad array of proteins present in biofluids. The limitation of proteins acting as biomarkers for certain diseases includes its rapid degradation by proteases in circulation and its relatively poor specificity. However, with the assistance of EVs, EVs could be identified by membrane proteins, which helps to tag enveloped proteins and protects them from lysis. As mentioned, one of the obstacles to elevating the survival rate of HNSCC patients is the early detection of cancer; therefore, many efforts have been made in this field with respect to exosomal proteins [175].

Liu et al. proposed that the detection of exosomal cyclophilin A (CYPA), a protein from immunophilin, helped distinguish NPC patients from healthy controls, which could greatly improve diagnostic precision when combined with the EBV antibody test [141]. You et al. explained that HS1-associated protein X-1 (HAX-1) was overexpressed in many human malignancies, such as HNSCC and especially NPC [176], and they also announced that exosome-packaged HAX-1 had a better performance when serving as a biomarker for LNM, clinical stage, and the prognosis of NPC [177].

The therapeutic effects of these immune checkpoint inhibitors (ICIs) targeting the PD-1/PD-L1 pathway led to survival benefits for platinum-pretreated recurrent and metastatic HNSCC patients [178]. The levels of PD-L1 expressed on the exosomes in circulation was proved to be correlated with patients’ UICC stage and LNM status [179]. Patients with higher expressions of PD-L1 exhibited longer DFS, and postoperative patients faced a higher risk of relapse or recurrence if they had a low PD-L1 expression status. PD-L1 was identified as the earliest indicator of treatment failure and prognosis prediction [179].

Likewise, Kannan et al. noticed that, in OPSCC, the exosomal proteins SIRPA and HPV-16-E7 were elevated in the serum of HPV-16-associated OPSCC [180]. Li et al. disclosed that the protein expression levels of MMP-1 significantly differed with the stratification of malignant potential; it only increased from MSC-derived exosomes collected from normal oral mucosa, oral leukoplakia, and OSCC [54]. These studies suggested that virus-derived and exosome-carried components and proteins could act as biomarkers for HNSCC [54,180].

## 4. EV as Prospective Biomarkers in HNSCC

Cancer-derived EVs have promising potential to be used as tumor biomarkers, not only for screening and diagnosis but also for monitoring cancer progression and the prediction of patients’ responses to therapy, which has been widely demonstrated in some cancers, such as non-small cell lung cancer [181,182,183]. However, EV detections and applications have not been widely adopted for clinical use since we are still pursuing a greater platform that could ensure highly sensitive and specific methods, but these require minimal sample volume for testing. Additionally, the platform should be capable of detecting different cargoes delivered by EVs (i.e., DNA, RNA, proteins), since they might also be specific biomarkers for certain diseases, and if a platform for detecting EVs could realize such a high-throughput analysis for development, then the clinical application for EVs as biomarkers would be applied in future [30].

The profiles of EV components between malignant tumor cells and normal cells vary significantly, and differences also exist between HNSCCs with and without specific virus infections; these ensure the significance of EVs as competitive candidate biomarkers for HNSCC diagnosis and prognosis.

The merits of EV detection in liquid biopsies in HNSCC include easy sampling, less invasiveness and high acceptance by patients. Compared to CTCs or cfDNA, the concentration of EVs is relatively higher, and EVs can be found in more kinds of biofluids, such as urine and sweat, not just in blood. Despite abundant studies supporting and discovering a great number of EV-conveyed components to be screening tools, one of the limitations that restrict wide clinical application is the interference caused by other endogenous substances and current EV-detection technologies; it is still challenging to identify and extract the tumor-derived components. EVs are likely to exhibit varying sensitivity and specificity due to different sample types and targeting substances, and there is still a lack of standardization of techniques for detection and isolation; these are all problems to solve before they are widely used. In addition, it is also a major obstacle to reaching a well-established quantified standard for tumor screening because the number of circulating EVs differs from person to person, and the abundance of EVs could be affected by confounding effects from nonmalignant cells, such as platelets and megakaryocytes, under certain stimuli. Unlike exosomes, the knowledge about microvesicles still remains occult, especially in head and neck cancer, which probably requires greater attention and effort. However, it is believed that the rapidly developing technologies will continuously and gradually improve the use of EVs as biomarkers in liquid biopsies.

Since it is difficult to set a defined range of values, the individual variance in EV numbers could be partly eliminated if we simply evaluate them case by case instead of comparing them to the mean or a certain range. For instance, the changes in these EV-containing cargoes could be used as indicators for distinguishing different stages of the disease, such as following tumor progression and observing effective treatment. When the goal is not to precisely discriminate patients from the normal population but to monitor the therapeutic effects of interventions, disease diagnosis and treatment, the advantages of liquid biopsy such as easy sampling and minimal invasiveness are easily demonstrable. However, as shown, the clinical trials to investigate EV assessments in disease monitoring are lacking, which requires further study (Table 1).

## 5. Conclusions

EVs, intricate structures that contain diverse components, such as DNA, RNA, and proteins, participate in many pivotal pathways of HNSCC. The tumor microenvironment and metabolic phenotype are greatly altered after receiving the message molecules delivered by EVs, which are released by connective tissue cells (such as cancer-associated fibroblasts and mesenchymal stem cells), as well as immune cells. The assistance of EVs facilitates the formation of pre-metastatic niches, activation of the vasculature, and fusion with parenchymal cells, which greatly promotes tumor metastasis. The cargoes transported by vesicles also influence the resistance to chemoradiotherapy, and it sheds light on the possibility that EVs can serve as therapeutic target or drug carriers. When compared to tissue biopsy, the advantages of EV detection are easy sampling and minimal invasiveness, which make it more acceptable by patients; when compared to cfDNA, CTC, or other detections based on liquid biopsy, EVs can be extracted from more diverse biofluids, and their membranous structure enables cargoes conveyed by EVs to be preserved more stably and detected even in small amounts. However, the clinical application and formal approval for EV assessment in the screening and diagnosis of HNSCC is restricted by the stagnant development of detection technologies and individual differences in terms of the abundance and expression of the tumor-specific components of EVs. There is a good prospect for their application in the prediction of the response to interventions and relapse or prognosis, since it focuses on the dynamic monitoring of the treatment process and the changes in patient conditions.

## Abbreviations

EV, extracellular vesicles; HNSCC, head and neck squamous cell carcinoma; DNA, deoxyribonucleic acid; RNA, ribonucleic acid; TNF, tumor necrosis factors; MAPK, mitogen-activated protein kinase; LNM, lymph node metastasis; EpCAM, epithelial cell adhesion molecule; PSA, prostate specific antigen; FFPE, formalin fixed paraffin-embedded.
